# α_1_-Acid Glycoprotein-Decorated Hyaluronic Acid Nanoparticles for Suppressing Metastasis and Overcoming Drug Resistance Breast Cancer

**DOI:** 10.3390/biomedicines10020414

**Published:** 2022-02-09

**Authors:** Haneen Omar, Roa’ Fardous, Yasser M. Alhindi, Alhassan H. Aodah, Mram Alyami, Mohammed S. Alsuabeyl, Waleed M. Alghamdi, Ali H. Alhasan, Abdulaziz Almalik

**Affiliations:** 1 National Center for Pharmaceutical Technology, Life Science and Environment Research Institute, King Abdulaziz City for Science and Technology (KACST), Riyadh 11564, Saudi Arabia; rsfardous@alfaisal.edu (R.F.); yalhindi@kacst.edu.sa (Y.M.A.); aaodah@kacst.edu.sa (A.H.A.); malsubyl@kacst.edu.sa (M.S.A.); wghamdi@kacst.edu.sa (W.M.A.); 2 KACST-BWH/Harvard Center of Excellence for Biomedicine, Joint Centers of Excellence Program, King Abdulaziz City for Science and Technology (KACST), Riyadh 11533, Saudi Arabia; 3 Pharmaceutical Analysis Lab at King Abdulaziz City for Science and Technology (PALab), Life Science and Environment Research Institute, King Abdulaziz City for Science and Technology (KACST), Riyadh 11533, Saudi Arabia; 4 Smart Hybrid Materials (SHMs) Laboratory, Advanced Membranes and Porous Materials Center, King Abdullah University of Science and Technology (KAUST), Thuwal 23955, Saudi Arabia; mram.alyami@kaust.edu.sa; 5 National Center for Biotechnology, Life Science and Environment Research Institute, King Abdulaziz City for Science and Technology (KACST), Riyadh 11533, Saudi Arabia; 6 College of Science and General Studies, Alfaisal University, P.O. Box 50927, Riyadh 11533, Saudi Arabia

**Keywords:** multidrug resistance (MDR), tumor metastasis, hyaluronic acid (HA) nanoparticles, α_1_-acid glycoprotein (AGP), breast cancer cells, immune-suppressing nanoparticles

## Abstract

Robust inflammation-suppressing nanoparticles based on α_1_-acid glycoprotein (AGP)-conjugated hyaluronic acid nanoparticles (AGP-HA NPs) were designed to regulate breast cancer cells’ sensitivity to chemotherapy and to suppress tumor metastasis. The successful conjugation between AGP and HA NPs was confirmed using FTIR, zeta potential, and UV–vis spectroscopy. In vitro studies on MCF-7 cells indicated the remarkable ability of AGP-HA NPs in suppressing migratory tumor ability by 79% after 24 h. Moreover, the efficacy study showed the high capability of AGP-HA NPs in modulating MDA-MB-231 cells and restoring cell sensitivity to the chemotherapeutic drug doxorubicin (DOX). Furthermore, the finding obtained by flow cytometry and confocal spectroscopy demonstrated that AGP-HA NPs enhanced DOX uptake/retention and aided it to reach cell nucleus within 4 h of incubation. Therefore, AGP-HA NPs represent a viable and effective treatment option to strengthen the anticancer effects of chemotherapeutic agents and potentially improve patients’ survival rates.

## 1. Introduction

Breast cancer is the most frequently diagnosed cancer worldwide and the leading cause of death among women [1]. Cancer treatment strategies, including surgery, chemotherapy, radiation therapy, and immunotherapy, have faced many clinical complexities over the last four decades affecting their therapeutic efficacy [2]. Chemotherapy is one of the most potent treatment modalities for solid tumors [3,4]; however, the emergence of multiple drug resistance (MDR) and metastatic characteristics often lead to chemotherapy resistance [5,6,7]. MDR is a phenomenon where tumor cells develop resistance to chemotherapeutic drug molecules, leading to a limit to their therapeutic efficacy. It has a complicated mechanism and can be mediated by various factors, greatly associated with drug efflux pumps belonging to the adenosine triphosphate (ATP)-binding cassette (ABC) transporter family, including P-glycoprotein (P-gp) [8,9]. Overexpression of P-gp can actively efflux numerous chemotherapeutic agents (ex. doxorubicin (DOX)) outside of the cell membrane, which decreases intracellular drug retention and subsequently reduces their efficacy [10], in addition to facilitating tumor metastasis [11]. Many strategies have been evolved to circumvent MDR in cancer chemotherapy, including P-gp inhibitors [12], RNA-silencing [13], and co-administration strategies [14]. However, their clinical administration is still hindered by low efficacy, high toxicity, intolerable side effects, and expensive cost [15,16].

The nanoparticle-based approach has shown high potential in manipulating the MDR phenotype and enhancing drug uptake and retention [17,18,19]. They emerged as an alternative strategy to tackle MDR by incorporating or attaching anticancer drugs to nanocarriers, which can enter cells via endocytosis and escape the effect of P-gp efflux pumps. Even though such approaches are considered promising in addressing MDR, they were rather ineffective in addressing the underlying inflamed tumors and the resultant metastasis [20]. Indeed, inflamed tumors exhibited pro-apoptosis phenotypes, leading to enhanced tumor survival via increasing both the extracellular and intracellular levels of promoting oxidative stress [21]. Moreover, inflamed tumors induce the activation of Nuclear Factor-κB (NF-κB), leading to the release of Tumor Necrosis Factor-alpha (TNF-α) and Transforming Growth Factor (TGF-β), promoting pro-apoptotic pathways [22] and cell survival and mediating epithelial–mesenchymal transition (EMT) and metastasis [20].

Recently, hyaluronic acid nanoparticles (HA NPs) have been reported to exhibit a remarkable feature by adsorbing a unique anti-inflammatory protein, α_1_- acid glycoprotein (AGP), on their surface upon mixing with human serum proteins [23,24]. AGP is known to suppress the overexpression of pro-inflammatory cytokines in the tumor microenvironment, including (TNF-α) and interleukins (IL-6), in which it is linked to drug resistance and metastasis [25,26,27], while HA is a naturally occurring ligand with a high binding affinity for the cluster of differentiation-44 (CD44) receptors [28]. Overexpression of the CD44 receptor on various cancer cells surfaces, including breast cancer (ex. MCF-7 and MDA-MB-231 cell lines), aids tumor migration and invasion [29]. Therefore, HA-modified NPs are promising CD44-targeted carriers for the preferential tumor accumulation of chemotherapeutic agents [30,31].

In the present work, we hypothesize that a targeting nanoplatform based on immobilizing AGP into HA NPs (AGP-HA NPs) can suppress the inflammation of tumor cells, leading to the modulation of its MDR, thereby reducing tumor migration. This approach suggested that AGP-HA NPs could inhibit tumor migration by dampening inflammation, leading to the resensitization of MDR cells (MDA-MB-231) to the chemotherapeutic drug (DOX) via enhancing DOX lethality towards drug-resistance breast cancer cells.

## 2. Materials and Methods

### 2.1. Materials

α1-acid glycoprotein (AGP) C.A.T. #(66455-27-4), PBS, high pure chitosan, and lipopolysaccharide (LPS) from *E. coli* 026:B6 were from Sigma (L8274-10MG). The QuantiPro TM BCA Assay kit was from Sigma Aldrich; St. Louis, MO, USA (QPBCA-1KA). High purity chitosan (deacetylation degree over 75–85% mol and viscosimetric molecular weight 50–190 kDa), sodium triphosphate pentabasic (TPP), 1 M hydrochloric acid (HCl), and sodium hydroxide (NaOH) were obtained from Sigma-Aldrich (Gillingham, UK). Milli-Q water was used in all synthetic experiments. Hyaluronic acid (HA; weight average molecular weight ≈200 kDa) was purchased from Medipol SA; Lausanne, Switzerland. Regenerated cellulose (RC) dialysis membrane (MWCO 10kDa) was obtained from Spectra Por, Spectrum Laboratories Inc.; Los Angeles, CA, USA.

### 2.2. Methods

Dynamic light scattering (DLS). Hydrodynamic diameter (Z-average size), polydispersity index (PDI), and zeta potential measurements were always performed at room temperature using a Zetasizer Nano ZS instrument (Model ZEN3600, Malvern Instruments Ltd.; Malvern, UK) fitted with a solid-state HeNe laser (λ = 633 nm) at a scattering angle of 173°. Scanning electron microscopy (SEM) was recorded by JSM-IT500HR InTouchScope. A nano spray dryer (Model B-90, BÜCHI Labortechnik AG, Flawil, Switzerland) was used as a dryer. The Millipore Labscale T.F.F. system (Millipore Corporation; Burlington, MA, USA) was used, along with a Pellicon XL TFF cassette with a 500 kDa molecular weight cut-off membrane (Millipore Corporation, Burlington, MA, USA), as an ultrafiltration technique. FTIR spectra were recorded using a Thermo Scientific spectrometer (Nicolet iS10). Absorption spectra were recorded using a Varian Cary 5000 UV–vis-NIR spectrophotometer. Powder XRD measurements were performed using a Panalytical X’Pert Pro X-ray powder diffractometer using Cu Kα radiation (40 V, 40 mA, λ = 1.54056 Å) in a θ–θ mode from 20° to 90° (2 θ).

### 2.2.1. Synthesis of CS-TPP NPs

A 0.069 wt.% chitosan solution was prepared by dissolving CS (69 mg) in 4.6 mM HCl solution (100 mL) and stirring overnight at room temperature. The pH was then adjusted to 5 by adding appropriate volumes of 0.5 M NaOH. A 0.1 wt.% TPP solution was prepared by dissolving TPP (10 mg) in deionized water (10 mL) and stirring for 30 min at room temperature, then the pH of the solution was adjusted to 5 using 0.5 M HCl. Both solutions were filtered through a 0.22 µm pore size filter; 7 mL of TPP solution was added to CS solution (93 mL) for a final volume of 100 mL, making a 9:1 CS:TPP mass ratio to produce 0.064 and 0.0071 wt.% of CS and TPP, respectively. Then, under magnetic stirring and agitation, complexation to form the CS NPs was carried for 30 min at room temperature. Finally, the dispersed NPs were dialyzed against deionized water (2 h, MWCO 10 kDa).

### 2.2.2. Synthesis of HA NPs

A 0.2 wt.% HA solution was prepared by dissolving HA (120 mg) in deionized water (60 mL) and stirring overnight at room temperature. Then, the solution was filtered through a 0.22 µm pore size filter; 50 mL of CS-TPP NPs solution was slowly added under vigorous stirring to an equal volume of HA solution and stirred for 30 min at room temperature. The dispersed NPs were then dialyzed against deionized water (2 h; MWCO 10 kDa). The produced NPs were then dried using Buchi’s nano spray dryer in the open-loop configuration, where inlet temperature was adjusted to 120 °C and both motor and spray power were set to 50%.

### 2.2.3. Synthesis of AGP-HA NPs

A 0.1 wt.% AGP solution was prepared by dissolving AGP (1 mg) in deionized water (1 mL). The volume ratio of NPs:AGP (1:3) was prepared with a final volume of 400 µL, where 300 µL of AGP (1 mg/mL) solution was added to 100 µL of HA NPs solution (1 mg/mL) for a final volume of 400 µL, making a 1:3 NPs:AGP volume ratio. Then, the solution was gently stirred for 96 h at room temperature. The sample was then filtered using Millipore’s Labscale T.F.F. System to remove unbounded AGP.

### 2.3. In Vitro Studies

#### 2.3.1. Cell Culturing

MDA-MB231 cells were obtained from the American Type Culture Collection (ATCC HTB-26MET, Manassas, VA, USA) and subsequently cultured in Dulbecco’s modified Eagle’s medium (DMEM) containing 10% fetal bovine serum (FBS) and 1% antibiotics (penicillin–streptomycin) at 37 °C in a humidified atmosphere containing 5% CO_2_. Briefly, MDA-MB-231 cells were seeded in a 96-well plate as 10 × 10^3^ cells/well and allowed to reach high confluency (~70%). After that, the culture medium was changed, and cells were incubated with different concentrations (50, 100, and 300 µg/mL) of AGP-HA NPs in 200 μL of opti-DMEM medium at 37 °C for 48/72 h. Cells not being exposed to AGP-HA NPs served as untreated control (UTC).

#### 2.3.2. Cell Viability Assay

To study cytotoxicity, AlamarBlue assay was performed according to the manufacturer’s protocol. Briefly, MDA-MB-231 cells were incubated with different concentrations (50, 100, and 300 μg/mL) of AGP-HA NPs for 48/72 h. As incubation completed, culture media were replaced with fresh media containing 10% of AlamarBlue reagent and incubated for 3–4 h at 5% CO_2_ and 37 °C. The UTC served as a negative control. Fluorescence spectra were recorded at λ_ex_: 570, λ_em_: 600 using BioTek Cytation 5™ multi-mode Microplate Reader-Gen5™ software.

#### 2.3.3. Scratch Assay

MCF-7 cell lines were seeded in a 96-well plate at 10 × 10^3^ cells/well and allowed to reach high confluency (~70%) prior to treatment. Wounds were made by scraping the cells with a P-200 pipette tip. Detached cells were washed away by PBS, and photos were immediately taken (time 0). Next, cells were treated with (10 µg/mL) LPS for 24 h. Then, the culture medium was changed, and cells were treated with 100 µg/mL HA NPs, 100 µg/mL AGP, and 100 µg/mL AGP-HA NPs. Untreated wells and LPS treated were used as controls. Cells were incubated for 24 and 48 h, and images were taken to monitor cells confluency in wounds by BioTek^®^ Cytation3.

#### 2.3.4. LPS Enhances the Invasive Potential in MDA-MB-231

LPS was suggested to promote cancer cell migration. In in vitro scratch assays, the cells were seeded in 12-well plates at a density of 100 × 10^3^ cells/well. After incubation overnight, cells were stimulated with LPS (5, 10, and 20 µg/mL) for 48 h and then scraped by a p20 pipette tip to create a straight-line cell-free scratch. Each well was washed with PBS three times to remove the remaining unattached cells and debris. The scratch area was marked and photographed in different time intervals (2, 4, 12, 24, and 48) h. The distances were measured by the software ImageJ, and cell motility was quantified by measuring the distance between the migrating cell boundaries. Data were analyzed statistically. Then, the concentration of (20 µg/mL) LPS was selected to enhance the invasiveness of MDA-MB-231 cells in the following studies.

#### 2.3.5. LPS Mediates DOX-Resistance Activation

MDA-MB-231 cells were seeded in a 96-well plate (10 × 10^3^ cell per well) and treated with or without LPS at a concentration of (20 µg/mL) for 24 h. Cells were then left untreated or treated simultaneously with (0.67 µg/mL) of the chemotherapeutic drug (DOX) and various doses of AGP-HA NPs (50, 100, and 300 µg/mL). After further incubation for 48 or 72 h, cells were subjected to AlamarBlue cell viability assay. As incubation completed, culture media were replaced with fresh media containing 10% of AlamarBlue reagent and incubated for 3–4 h at 5% CO_2_ and 37 °C. The UTC served as a negative control, and LPS-stimulated/nonstimulated control groups were untreated with nanoparticles. Fluorescence spectra were recorded at λ_ex_: 570, λ_em_: 600 using BioTek Cytation 5™ multi-mode Microplate Reader-Gen5™ software.

#### 2.3.6. Confocal Fluorescence Microscopy

For confocal fluorescence microscopy observation, MDA-MB-231 cells were seeded onto an 8-well chamber slide at a density of 10 × 10^3^ per well for 48 h. The cells were subsequently incubated with 200 µL LPS in optiDMEM at a concentration of (20 µg/mL) for 24 h. Media was then discarded, and a culture medium containing (0.67 µg/mL) free DOX and (50 µg/mL) AGP-HA NPs + DOX were added into each well for 1, 2, 3, and 4 h at 5% CO_2_ and 37 °C. The cells were then washed three times with PBS. Nuclei were stained with DAPI for 10 min at 37 °C and then washed three times with PBS to remove the excess of the dye. Transfected cells were fixed with 4% paraformaldehyde and photographed by CLSM (Zeiss LSM 710 inverted confocal microscope).

#### 2.3.7. Flow Cytometry

In order to examine the internalization of the drug into the cells, fluorescence-activated cell sorting (FACS) was carried out. MDA-MB-231 cells were sub-cultured on 24 well plates at a density of 100 × 10^3^ cells per well for 48 h. Then, the cells were treated with LPS at a concentration of (20 µg/mL) for 24 h. Free DOX (0.67 µg/mL) and (50 µg/mL) AGP-HA NPs + DOX were incubated with MDA-MB-231 cells for 1, 2, 3, and 4 h at 5% CO_2_ and 37 °C. After removing the medium, the cells were washed three times with cold PBS and harvested using trypsin solution and gently suspended in 2 mL fresh medium. The uptake efficiency of the cells was analyzed by flow cytometry (BD LSRFortessa) with CellQuest software.

#### 2.3.8. Statistical Analysis

All experiments were performed at least in independent triplicate. Error bars in the graphical data represent standard deviations. Comparisons between different groups were made using the Student *t*-test. Statistical significance was defined as *** *p* < 0.001, ** *p* < 0.01, and * *p* < 0.05.

## 3. Results and Discussion

**Scheme 1:**
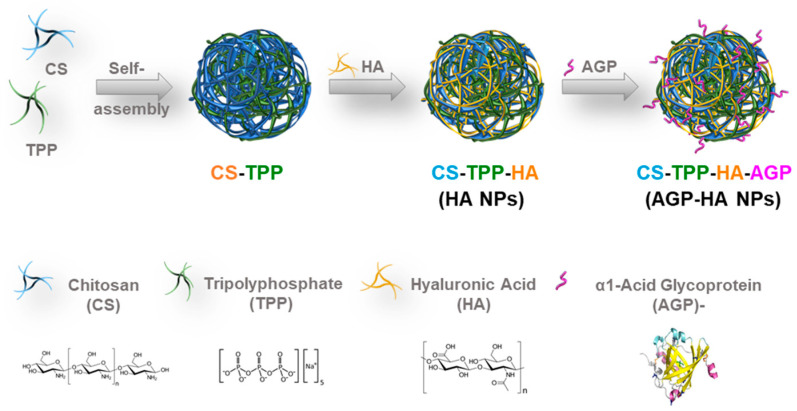
Synthesis of α_1_-acid glycoprotein (AGP)- hyaluronic acid nanoparticles (AGP-HA NPs).

Typically, HA NPs are prepared by following the conventional ionic gelation method [32]. Initially, CS-TPP NPs were prepared based on the inter- and intra-molecular ionic linkages created between the negatively charged phosphate groups of pentasodium tripolyphosphate (TPP) and the positively charged amino groups of chitosan (CS). Subsequently, CS-TPP NPs were added to the HA solution to yield HA NPs in which hydrogen bonding and electrostatic attractions took place between the carboxylic groups of HA and the amine groups of CS. The produced NPs were then dried using the nano-spray drying technique to yield powder NPs in order to maximize both the yield and the quality of dispersion when re-solubilized in solution. Eventually, the dried NPs were redispersed and incubated with the AGP solution to yield AGP-HA NPs (Scheme 1).

### 3.1. Characterization of AGP-HA NPs

**Figure 1:**
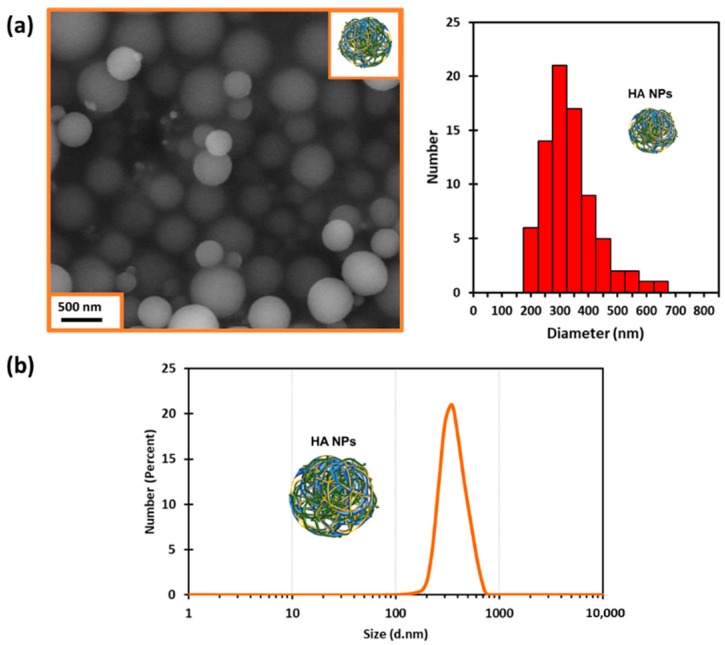
(**a**) SEM images of hyaluronic acid nanoparticles (HA NPs) and their particle size distribution, (**b**) DLS analysis of hyaluronic acid nanoparticles (HA NPs) was plotted by the number of nanoparticles as a function of their hydrodynamic diameter.

The size and the morphology of the HA NPs were then assessed via scanning electron microscopy (SEM) (Figure 1), revealing spherically shaped NPs with an average particle size of 320 nm. The size distribution of the NPs was confirmed quantitatively using dynamic light scattering (DLS) measurements, which displayed a single population of NPs with an average hydrodynamic diameter of 352 ± 22 nm (Figure 1b).

The X-ray diffraction (XRD) patterns of CS displayed two distinct diffraction peaks at 2 θ = 10° and 20°, corresponding to the crystallographic planes (020) and (110), as shown in Figure S2. After TPP addition, the XRD peaks of CS reduced, suggesting the crosslinking with TPP to form CS-TPP NPs [33,34]. For HA NPs, the XRD peaks were enhanced compared to CS-TPP NPs due to the interactions between the amino groups of CS with carboxyl groups of HA, which limited the movement of the CS chain and reduced its crystallization.

**Figure 2:**
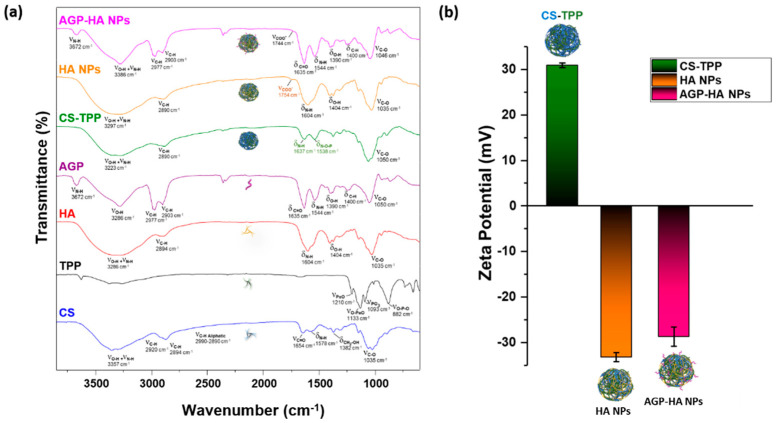
(**a**) FT-IR spectra and (**b**) zeta potential of chitosan-pentasodium tripolyphosphate (CS-TPP), hyaluronic acid nanoparticles (HA NPs), and α_1_-acid glycoprotein-conjugated hyaluronic acid nanoparticles (AGP-HA NPs) and their separate component, confirming the successful incorporation of hyaluronic acid nanoparticles (HA NPs) components and the integration of α_1_-acid glycoprotein (AGP) with hyaluronic acid nanoparticles (HA NPs).

The successful interaction between CS chains and TPP was demonstrated by FTIR spectroscopy, as evident from the stretching vibrations of O-H and N-H bands that became wider compared to those of the native CS and TPP crosslinker, indicating an enhancement of the hydrogen bonding interactions (Figure 2a). Additionally, the shift in the peak of -NH bending vibration from 1578 to 1637 cm^−1^ was attributed to the interaction between the amino group of CS and the phosphate anion of TPP [35]. Moreover, the appearance of a new peak at 1538 cm^−1^, assigned to the N-O-P stretching vibration, indicates that TPP anions were crosslinked successfully with the amino groups of CS to form CS-TPP NPs through the ionotropic gelation method [36,37]. A new shoulder peak at 1754 cm^−1^, corresponding to the protonated acidic groups of HA (COOH), appeared in the HA NPs spectrum, confirming the successful incorporation of HA with CS-TPP NPs, in addition to the presence of the characteristic peaks of CS and HA [38,39]. Reduction in the stretching frequency of all peaks due to the hydrogen bonding between the protein and the NPs indicates the integration of the AGP protein with HA NPs.

The zeta potential of the NPs was consistently reversed from +31 ± 0.5 to −33 ± 1 mV after the addition of HA due to the complexation of HA molecules onto the surface of the CS-TPP NPs. A further change occurs in zeta potential value after the decoration of HA NPs with AGP protein, where the zeta potential increased from −33 ± 1 to −28 ± 2 mV, as shown in Figure 2b. This rise in zeta potential value could be explained by the partial shielding of the NP surface by AGP proteins. In addition, the UV–vis spectroscopy exhibited a blue shift in the UV–vis spectrum of HA NPs when decorated with AGP compared to free AGP (Figure S3). This shift indicates an interaction between the HA NPs and AGP, which is attributed to π→π* transitions in the peptide bonds of AGP [40], and thus verified the successful interaction between the HA NPs and AGP.

### 3.2. AGP-HA NPs Inhibit MCF-7 Breast Cancer Cell Migration

**Figure 3:**
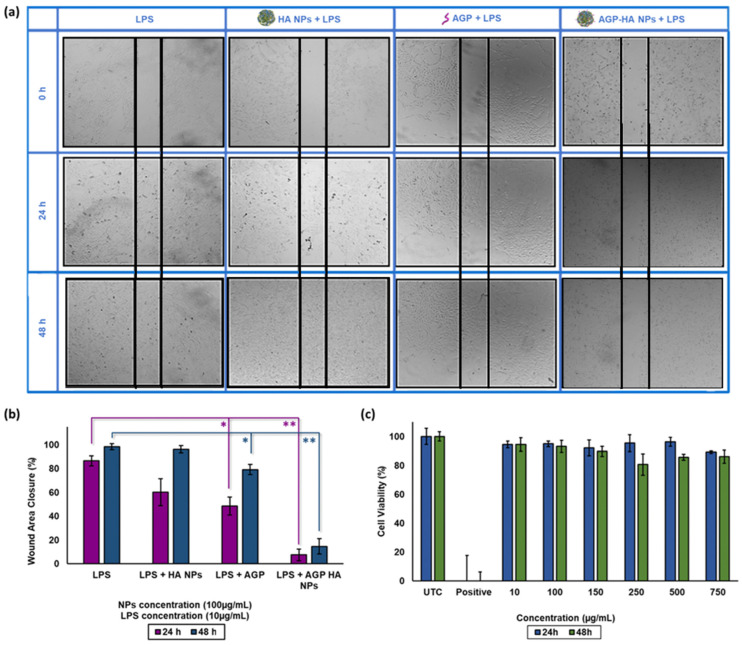
(**a**,**b**) Scratch assay of hyaluronic acid nanoparticles (HA NPs), α_1_-acid glycoprotein (AGP), α_1_-acid glycoprotein-conjugated hyaluronic acid nanoparticles (AGP-HA NPs) at 24 and 48 h incubation time mediates with LPS in MCF-7 cells. (**c**) Cytotoxicity of hyaluronic acid nanoparticles (HA NPs) at 24 h and 48 h incubation time using AlamarBlue assay in MCF-7 cells. All data presented in averages and standard deviations from at least three independent experiments. Statistical analysis was determined using Student’s *t*-test ( ** *p* < 0.01, * *p* < 0.05).

The biocompatibility of AGP-HA NPs was evaluated by AlamarBlue assays in breast cancer MCF-7 cells at 24 and 48 h incubation times with varied concentrations. Figure 3c illustrates that NPs were nontoxic toward MCF-7 cells up to 750 µg/mL, supporting the notion that AGP-HA NPs possess excellent biocompatibility.

Cancer cell migration and invasion play a crucial role in cancer metastasis. Scratch assays were performed using MCF-7 cells, which are known to express a high level of CD44 receptors. [41] In short, MCF-7 cells were treated with LPS (10 µg/mL) for 24 h to boost tumor migration and metastasis [42]. Afterward, AGP-HA NPs (100 µg/mL) were incubated further for another 24 and 48 h. As shown in Figure 3a,b, AGP-HA NPs significantly inhibited the migratory ability of MCF-7 cells by 79 ± 1% for 24 h and 83 ± 3% for 48 h compared to control groups, thus indicating the high capability of AGP-HA NPs in suppressing cell migration.

### 3.3. LPS-Mediated Migration in MDA-MB-231 Aggressive Breast Cancer Cells

Pro-inflammatory factors and chemotactic cytokines are key players in mediating chemotherapeutic resistance, cell proliferation, and metastasis in human cancers [43,44]. In order to deepen our evaluation of the synthesized NPs system, we used the aggressive MDA-MB-231 human breast cancer cell line for further studies. We utilized LPS to enhance the invasiveness of MDA-MB-231 cells, followed by an in vitro scratch assay over 48 h intervals. Altered cell proliferation and migration were apparent in response to LPS exposure at 20 µg/mL concentration after 24 h compared to the untreated control (Figure S4a). As revealed in Figure S4a,b, LPS-treated cells significantly migrated three times faster than the untreated ones. Such results confirm the capability of LPS to increase the invasiveness of aggressive breast cancer cells by about 41%.

### 3.4. AGP-HA NP Modulation of DOX Resistance in MDA-MB-231 Cells

**Figure 4:**
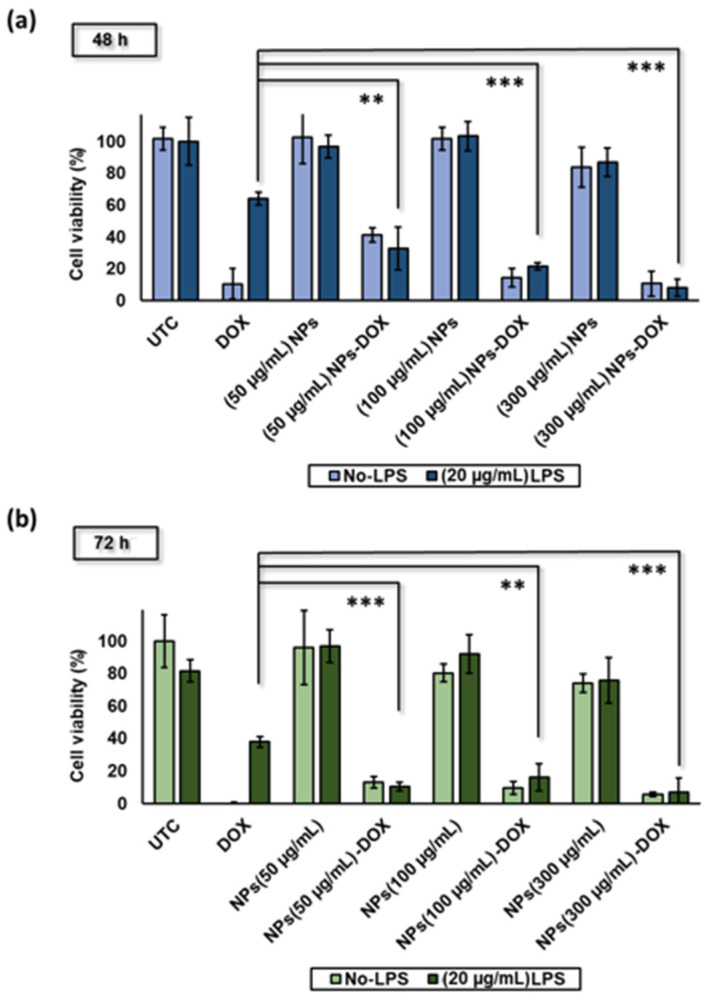
(**a**,**b**) Cytotoxicity of α_1_-acid glycoprotein-conjugated hyaluronic acid nanoparticles (AGP-HA NPs) at 48 h and 72 h incubation time using a CCK-8 kit, and LPS mediates DOX-resistance in MDA-MB-231 cells at various concentrations of α_1_-acid glycoprotein-conjugated hyaluronic acid nanoparticles (AGP-HA NPs) (50, 100, and 300 µg/mL) at 48 and 72 h incubation time All data presented in averages and standard deviations from at least three independent experiments. Statistical analysis was determined using Student’s *t*-test (*** *p* < 0.001, ** *p* < 0.01).

To start with, we first evaluated the efficacy of LPS in modulating the DOX resistance of MDA-MB-231 and inducing the MDR phenotype. Results showed that nonstimulated MDA-MB-231 cells were sensitive to DOX treatment at a concentration of 0.67 µg/mL, where DOX significantly reduced cell viability down to 11 ± 10% (Figure 4a). On the contrary, DOX failed to induce similar apoptosis efficacy in cells pre-treated with LPS (20 µg/mL) as a model for the MDR phenotype, with 64 ± 4% cell viability. As a result, pretreatment with LPS for 24 h was enough to achieve a 6-fold reduction in DOX chemosensitivity and cytotoxicity. The findings are consistent with previous reports of LPS-mediated MDR and the resultant DOX resistance [45].

To investigate the influence of AGP-HA NPs on modulating MDR, we first evaluated the cytotoxicity of AGP-HA NPs toward MDA-MB-231 cells at 48 and 72 h incubation time points. As shown in Figure 4a,b, AGP-HA NPs showed high biocompatibility, maintaining cell viabilities at 96 ± 23% and 80 ± 6% for 72 h at concentrations of 50 and 100 µg/mL, respectively. Following that, we measured the sensitivity of MDR cells to chemotherapeutic drugs (DOX) in the presence of AGP-HA NPs. Results showed potent cell growth-inhibitory effects when treating cells with DOX in the presence of AGP-HA NPs as opposed to DOX alone (Figure 4a,b). A dose-dependent efficacy was achieved when varying the concentrations of NPs. Cell growth-inhibitory effect dramatically increased with increases in the concentration of AGP-HA NPs, demonstrating the restoration of cell sensitivity to chemotherapy, thereby overcoming the MDR phenotype. Our described approach elucidates the indispensable role of AGP-decorated NPs in enhancing the efficacy of DOX against MDR aggressive breast cancer cells.

### 3.5. Cellular Uptake and Localization of the AGP-HA NPs + DOX in MDA-MB-231 Cells

**Figure 5:**
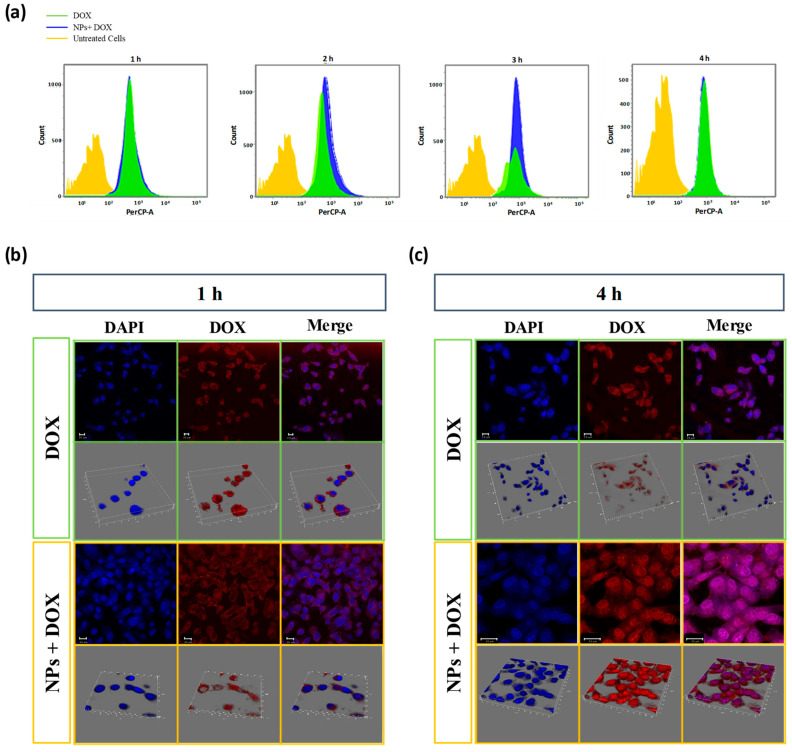
(**a**) Flow cytometry analysis of cellular uptake of α_1_-acid glycoprotein-conjugated hyaluronic acid nanoparticles (AGP-HA NPs) + DOX and free DOX in MDA-MB-231 cells incubated for 1, 2, 3, and 4 h. (**b**,**c**) CLSM images of α_1_-acid glycoprotein-conjugated hyaluronic acid nanoparticles (AGP-HA NPs) + DOX and free DOX in MDA-MB-231 cells incubated for 1 h (**b**) and 4 h (**c**). Nuclei were stained in blue with DAPI dyes, and DOX fluorescence in cells is red.

To induce tumor cell death efficiently, DOX should be delivered to the tumor cell nucleus, where it can induce DNA breakage. Herein, the intracellular localization of free DOX and AGP-HA NPs + DOX in MDA-MB-231 cells was studied using confocal laser scanning microscopy (CLSM) and flow cytometry analysis at different incubation time points. A 50 μg/mL concentration of AGP-HA NPs was selected as the lowest concentration to slow down the cellular uptake and retention rate of DOX while studying the internalization of DOX. As shown in Figure 5a, after 2 h of incubation, AGP-HA NPs + DOX exhibited a higher cellular uptake than free DOX, suggesting that NPs enhanced the amount of drugs associated with the cells. On the contrary, free DOX takes up to 4 h to reach the same fluorescence intensity as AGP-HA NPs + DOX.

The fluorescent images in Figure 5b,c present the comparison of intracellular DOX distribution after free DOX and AGP-HA NPs + DOX were incubated with MDA-MB-231 cells. After 1 h incubation with AGP-HA NPs + DOX, the fluorescence signal of DOX molecules was mainly distributed in the cell’s cytoplasm; a similar result was observed for free DOX (Figure 5b). Over time, the AGP-HA NPs + DOX showed a significant red fluorescence enhancement in the cytoplasm compared to the free DOX (Figure S5). Thus, this suggests that AGP-HA NPs enhanced DOX accumulation inside the cells by improving the intracellular DOX retention, presumably via bypassing DOX efflux in drug-resistant cells.

Noticeably, after 4 h post-incubation with free DOX, no DOX was observed in the nucleus and the fluorescence intensity is mainly from the cytoplasm (Figure 5c). This result was expected because of the efflux effect of P-gp in the MDA-MB-231 cells. In contrast, the situation was altered when the MDA-MB-231 cells were treated with AGP-HA NPs + DOX, where an abundant amount of DOX molecules were observed in both cell nuclei and cell cytoplasm.

The significant enhancement in the intracellular DOX accumulation and retention results could be attributed to the active targeting of AGP-HA NPs towards MDA-MB-231 cells and the anti-inflammatory properties of AGP protein. CD44-mediated cell uptake followed by inflammation suppression could reduce the P-gp-mediated drug efflux, leading to improved intracellular drug retention. Consequently, DOX accumulation in the nucleus could enhance its action of intercalating DNA conducive to cytotoxicity.

## 4. Conclusions

In conclusion, we have developed a new inflammation-suppressing NP to inhibit tumor cell metastasis and reverse the MDR phenotype in breast cancer cells. The morphology, composition, and structure of the synthesized nanosystem were extensively studied, along with their safety profiles, using different techniques. In vitro studies exhibited a remarkable inhibition in the tumor migration of MCF-7 cells using AGP-HA NPs. Cellular uptake and cytotoxicity assays showed an improvement in DOX efficacy in the presence of AGP-HA NPs, resulting in a boost of the cytotoxicity of MDA-MB-231 cells. Moreover, flow cytometry and confocal microscopy verified the enhancement in DOX internalization and retention. AGP-HA NPs helped DOX to reach the cell nucleus and thus inhibit cell proliferation and induce apoptosis. Therefore, AGP-HA NPs provide a promising nanotherapeutic platform against MDR and metastatic tumors, where the tumor can be weakened via inflammation suppression prior to chemotherapy. This approach can be potentially applied to a wide range of medical challenges suffering from uncontrolled inflammatory complications, ranging from treating organ transplant rejection to autoimmune diseases.

## Data Availability

Not applicable.

## Supplementary Materials

The following supporting information can be downloaded at: https://www.mdpi.com/article/10.3390/biomedicines10020414/s1, Figure S1: DLS analysis of chitosan-pentasodium tripolyphosphate (CS-TPP) and hyaluronic acid nanoparticles (HA NPs).; Figure S2. XRD pattern of chitosan-pentasodium tripolyphosphate (CS-TPP), hyaluronic acid nanoparticles (HA NPs).and their separate component demonstrating the incorporation of HA with CS-TPP to form HA NPs.; Figure S3. UV-VIS spectrum of free AGP, hyaluronic acid nanoparticles (HA NPs) and α1-acid glycoprotein-conjugated hyaluronic acid nanoparticles (AGP-HA NPs), verifying the successful interaction between the HA NPs and AGP.; Figure S4. (a) LPS-concentration effect on the invasive potential of MDA-MB-231 cells at different LPS concentrations (5, 10 and 20 µg/mL) and different time intervals (0, 4, 12, 24 and 48 h), (b) the cell imaged using Cytation 5™ multi-mode Microplate Reader-Gen5™ software at 0, 24 and 48 h time intervals.; Figure S5. CLSM images of α1-acid glycoprotein-conjugated hyaluronic acid nanoparticles (AGP-HA NPs) + DOX and free DOX in MDA-MB-231 cells incubated for 1, 2, 3 and 4 h.
