# Supplementary material for: α1-Acid Glycoprotein-Decorated Hyaluronic Acid Nanoparticles for Suppressing Metastasis and Overcoming Drug Resistance Breast Cancer
Source: Biomedicines. 2022 Feb 9;10(2):414. doi: 10.3390/biomedicines10020414 (PMC8962395; doi:10.3390/biomedicines10020414)
Supplement: Supplementary file 1 [file biomedicines-10-00414-s001.zip › biomedicines-1513984-supplementary.pdf]

biomedicines

MDPI

Supplementary Materials

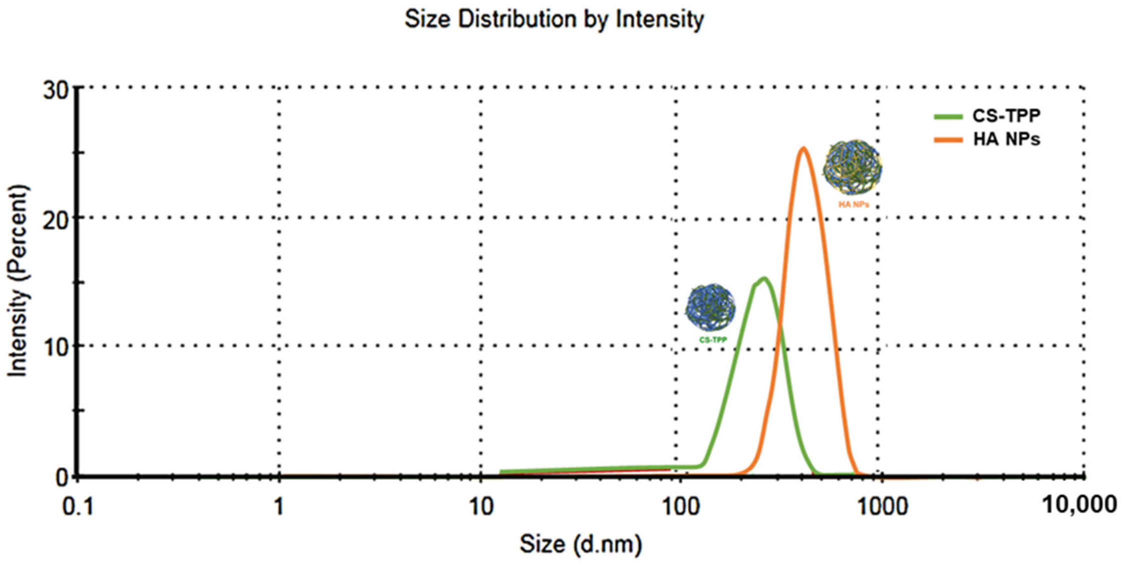

Figure S1. DLS analysis of chitosan-pentasodium tripolyphosphate (CS-TPP) and hyaluronic acid nanoparticles (HA NPs).

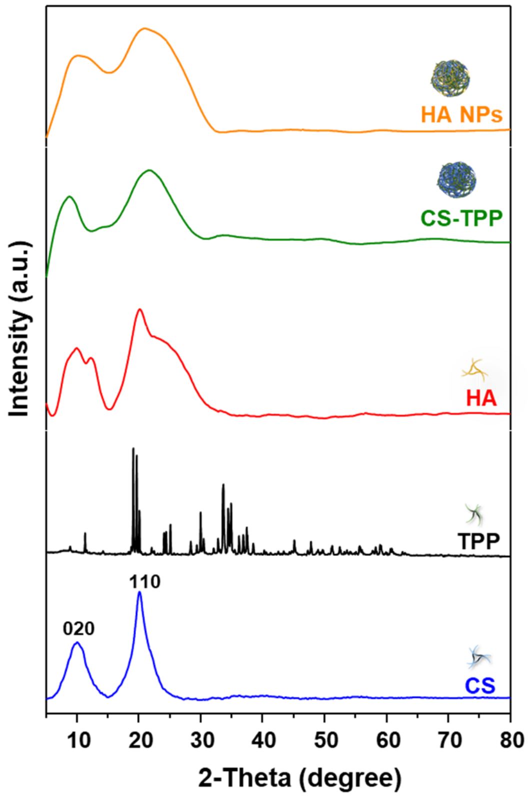

Figure S2. XRD pattern of chitosan-pentasodium tripolyphosphate (CS-TPP), hyaluronic acid nanoparticles (HA NPs).and their separate component demonstrating the incorporation of HA with CS-TPP to form HA NPs.

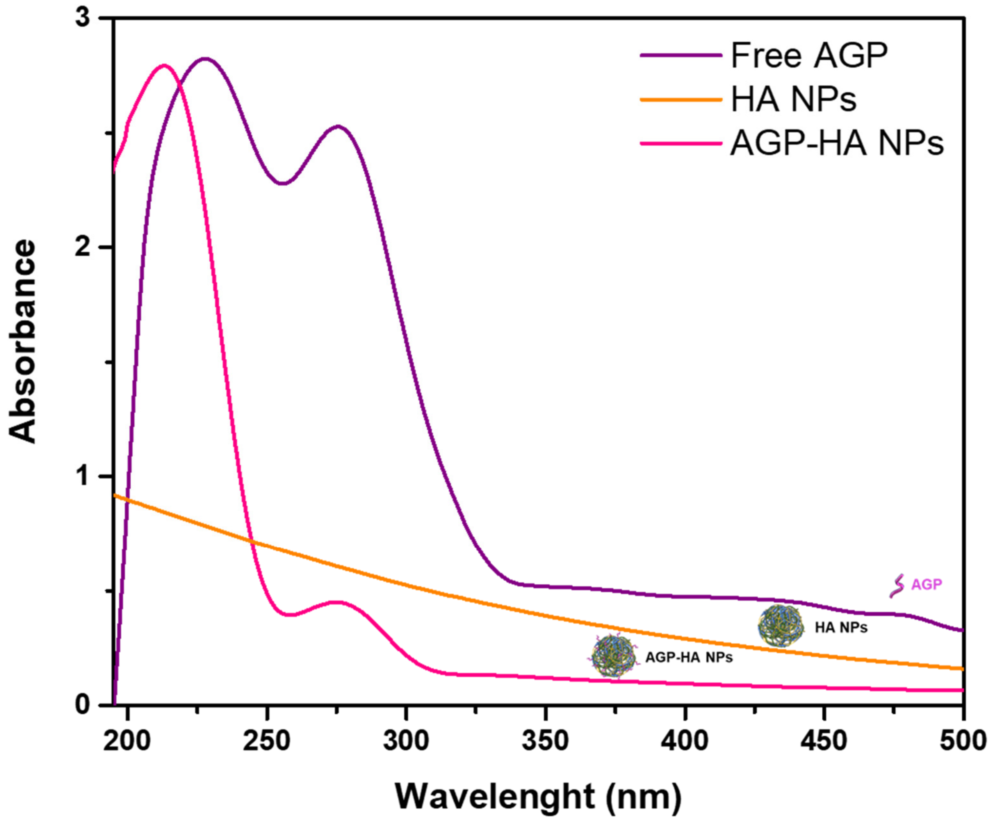

Figure S3. UV-VIS spectrum of free AGP, hyaluronic acid nanoparticles (HA NPs) and α_1-acid glycoprotein-conjugated hyaluronic acid nanoparticles (AGP-HA NPs),verifying the successful interaction between the HA NPs and AGP.

(a)

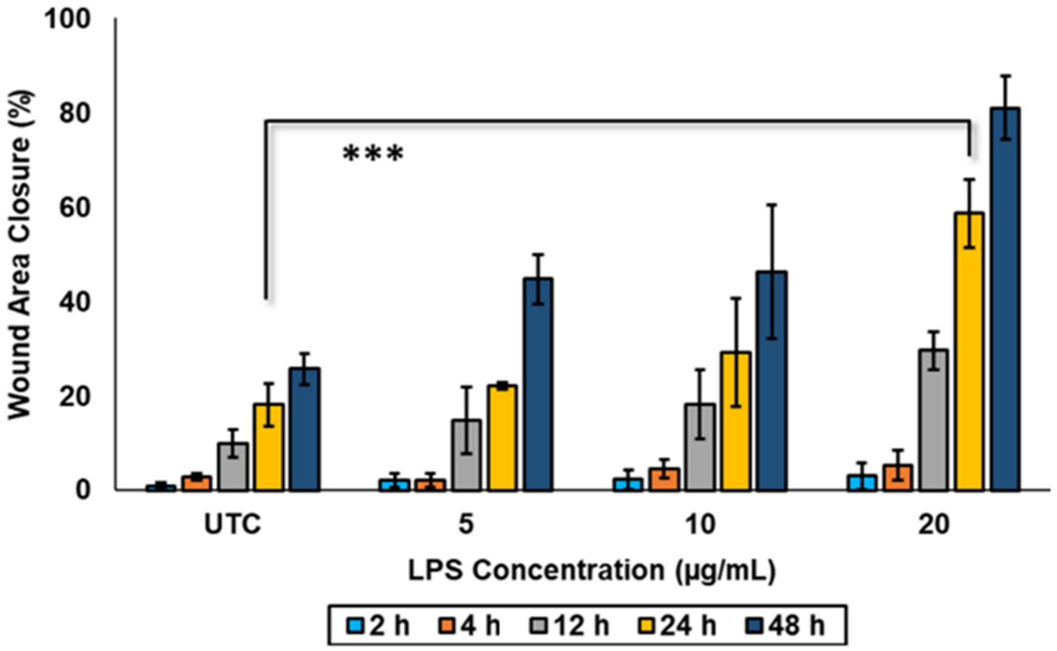

(b)

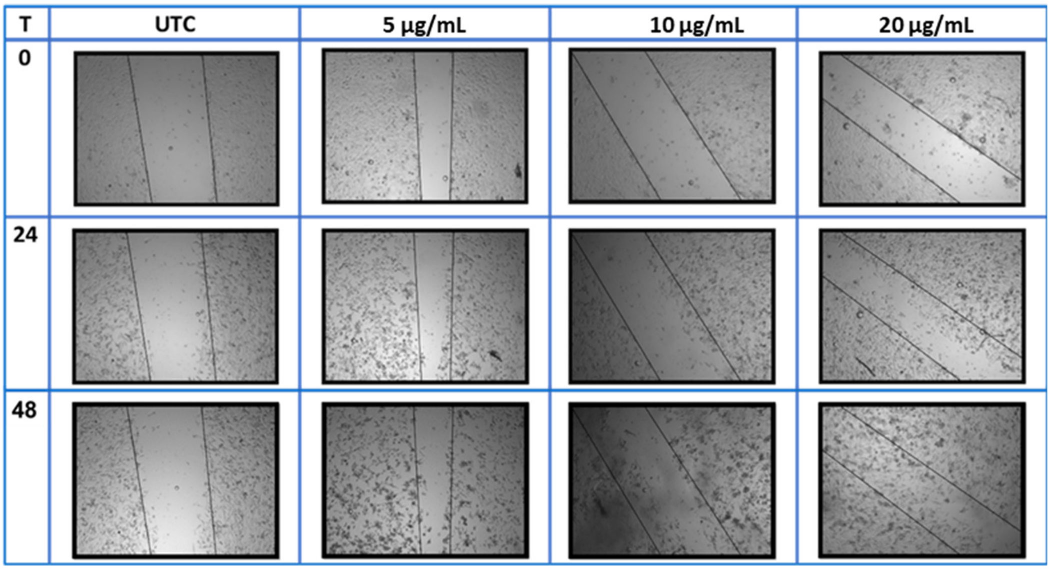

Figure S4. (**a**) LPS-concentration effect on the invasive potential of MDA-MB-231 cells at different LPS concentrations (5, 10 and 20 µg/ml) and different time intervals (0, 4, 12, 24 and 48 h), (**b**) the cell imaged using Cytation 5™ multi-mode Microplate Reader-Gen5™ software at 0, 24 and 48 h time intervals. Statistical analysis was determined using Student's *t*-test (***$p < 0.001$)

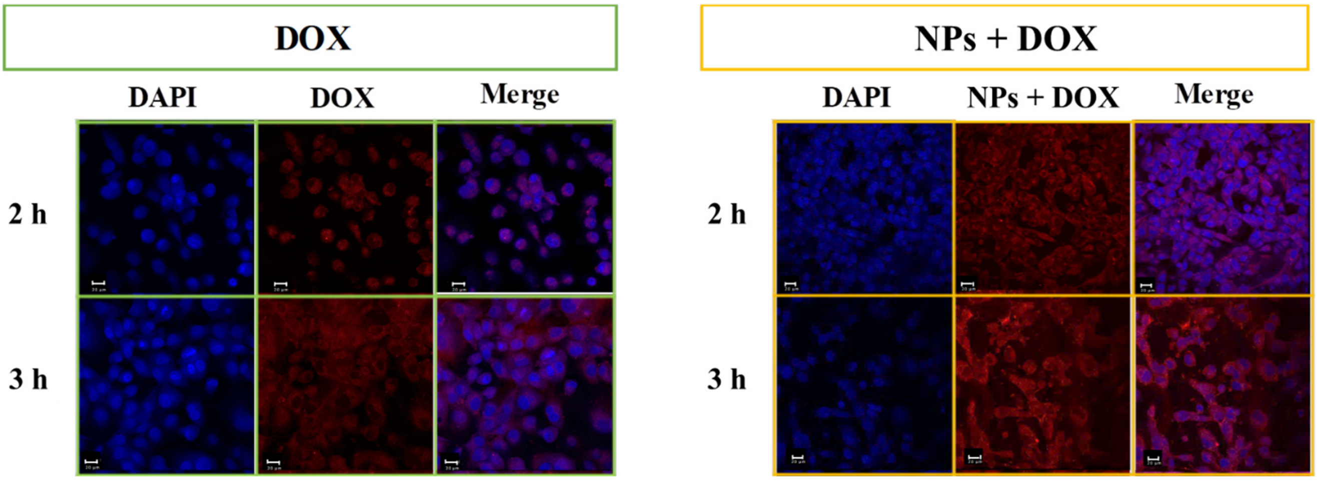

Figure S5. CLSM images of α_1-acid glycoprotein-conjugated hyaluronic acid nanoparticles (AGP-HA NPs) + DOX and free DOX in MDA-MB-231 cells incubated for 1, 2, 3 and 4 h. Nuclei were stained in blue with DAPI dyes, and DOX fluorescence in cells is red.
